# Priority concerns for people with intellectual and developmental disabilities during the COVID-19 pandemic

**DOI:** 10.1192/bjo.2020.122

**Published:** 2020-10-29

**Authors:** Sam Tromans, Michael Kinney, Verity Chester, Regi Alexander, Ashok Roy, Josemir W. Sander, Harry Dudson, Rohit Shankar

**Affiliations:** Leicestershire Partnership NHS Trust, UK; and Department of Health Sciences, University of Leicester, UK; Belfast Health and Social Care Trust, UK; Hertfordshire Partnership University NHS Foundation Trust, UK; and Norwich Medical School, University of East Anglia, UK; Hertfordshire Partnership University NHS Foundation Trust, UK; and University of Hertfordshire, UK; Coventry and Warwickshire Partnership NHS Trust, UK; and University of Warwick, UK; UCL Queen Square Institute of Neurology, UK; Chalfont Centre for Epilepsy, UK; and Stichting Epilepsie Instellingen Nederland (SEIN), the Netherlands; Department of Health Sciences, University of Leicester, UK; Cornwall Partnership NHS Foundation Trust, UK; and University of Exeter Medical School, Truro, UK

**Keywords:** COVID-19, intellectual disability, developmental disability, autism

## Abstract

**Background:**

The approach taken to support individuals during the coronavirus disease 2019 (COVID-19) pandemic needs to take into account the requirements of people with intellectual disabilities and/or autism, who represent a major vulnerable group, with higher rates of co-occurring health conditions and a greater risk of dying prematurely. To date, little evidence on COVID-related concerns have been produced and no report has provided structured feedback from the point of view of people with intellectual disabilities and/or autism or of their family/carers.

**Aims:**

To provide systemised evidence-based information of the priority concerns for people with intellectual disabilities and/or autism regarding the COVID-19 pandemic.

**Method:**

Senior representatives of major UK-based professional and service-user representative organisations with a stake in the care of people with intellectual disabilities and/or autism were contacted to provide a list of concerns across three domains: ‘mental health and challenging behaviour’, ‘physical health and epilepsy’ and ‘social circumstances and support’. The feedback was developed into statements on frequently reported priorities. These statements were then rated independently by expert clinicians. A video-conference meeting to reconcile outliers and to generate a consensus statement list was held.

**Results:**

Thirty-two organisations were contacted, of which 26 (81%) replied. From the respondent's data, 30 draft consensus statements were generated. Following expert clinician review, there was initially strong consensus for seven statements (23%), increasing to 27 statements (90%) following video conferencing.

**Conclusions:**

These recommendations highlight the expectations of people with intellectual disabilities and/or autism in the current pandemic. This could support policymakers and professionals’ deliver and evidence person-centred care.

Coronavirus disease 2019 (COVID-19), first recognised in December 2019, has led to a global pandemic posing a massive challenge for public health, clinical research and medical professionals.^1,2^ There is a clear need for a disability-inclusive response to COVID-19^3^ and historically people with intellectual and developmental disabilities (IDD), which includes people with autism, have been underserved in public health research initiatives.^4^ People with IDD have higher rates of comorbidities associated with a poorer outcome from COVID-19 infection, including diseases of the respiratory system, circulatory system and endocrine, nutritional and metabolic diseases, and are also at greater risk of dying from infection, particularly in younger age groups.^5^ There is a clear need for authorities to provide regular, transparent and accessible data on COVID-19 in people with IDD, and what such data means.^6^

Given the existing vulnerability of people with IDD, and their risk of morbidity and mortality from COVID-19, concerns specific to this population need to be identified. We aim to establish evidence-based information on the priority concerns for people with IDD during the COVID-19 pandemic, via liaison with stakeholder groups working with this population.

## Method

The methods described here were adapted from a study focusing on maintaining the safety of people with epilepsy during the COVID-19 pandemic.^7^ A STROBE checklist has been completed and submitted as it is a cross-sectional study.

Priority concerns from the IDD community were collected through key individuals within organisations and groups. The organisations were determined through discussion among the researchers. Of the organisations contacted, 29 were based in England (6 of which did not partake), as well as 2 in Ireland and 1 in Wales. The groups included a range of professionals and experts by experience, including healthcare professionals and carers of service users. They were asked to report the five highest ranking concerns pertaining to the COVID-19 pandemic in each of the following three domains:

(a) mental health and challenging behaviour;
(b) physical health and epilepsy; and
(c) social circumstances and support.

Based on the responses, ten statements relating to the most frequently occurring priorities were formulated for each of the three domains. These priorities were then rated by senior clinicians (R.A., A.R., R.S. and J.W.S.) on a scale of –10 (strongly disagree) to +10 (strongly agree). As was the case for the methodology previously described^7^ recommendations rated by all clinicians as ≥+7 during the first round of rating were determined to have attained a strong consensus. A video-conference was held between the authors of this paper to discuss further statements for which consensus was lacking, until a list of statements achieving strong consensus was reached or if not obtained the item was not included in the final priority statement.

### Ethics and participation consent

No ethical permission was required as this was done to gain knowledge and attitudes as part of a consensus statement. Further, it was with a group where consent was implicit by participation. All participants were advised at the start that participation was voluntary and their replies i.e. data would be analysed anonymously. We also used the NHS Health research authority tool (http://www.hra-decisiontools.org.uk/research/index.html), which confirmed that no ethical approval was required.

## Results

Thirty-two organisations were contacted, of which 26 (81%) responded; Appendix 1 details a list of organisations who responded to the request. The total number of priority concerns received was 118 for mental health and challenging behaviour, 116 for physical health and epilepsy and 105 for social circumstances and support. From the respondent's data, 30 draft consensus statements were generated, based on the most frequently recurring responses. These draft statements were rated by the expert panel (A.R., R.A., R.S. and J.W.S.). Following independent consensus statement ratings by the expert panel, a strong consensus for seven statements (23%) was obtained, which increased to 27 statements (90%) following statement discussion and revision via video-conference. The resultant 27 statements for which a strong consensus was attained are detailed below.

For the domain mental health and challenging behaviour the priority statements were as follows.

(a) Access to mental health services.
(b) Relapse of, or further deterioration in, mental health.
(c) Risk of disruption in the usual routines of people with IDD.
(d) Relapse of, or further deterioration in, challenging behaviours.
(e) Carer strain.
(f) Over-prescription of medication.
(g) Risk of difficulties in understanding COVID-19 and/or social distancing guidance.
(h) Risk of misattribution of symptoms and behaviours to IDD.
(i) Risk of difficulties in communication with professionals (for example personal protective equipment, video/phone consultations).

For the domain physical health and epilepsy the priority statements were as follows.

(a) Access to physical health services.
(b) Risk of physical inactivity due to lack of professional support in undertaking exercise.
(c) Risk stratification of individuals who need shielding.
(d) Risk of delayed presentation to services to address physical health complaints.
(e) Risk of physical complications from possible COVID-19 infection.
(f) Risk from not monitoring epilepsy-related concerns, including seizures and seizure-related injuries.
(g) Lack of physical health reviews and access to physical investigations.
(h) Risk of discrimination in end-of-life care and decision-making, including advanced care planning.
(i) Risk of lack of availability of personal protective equipment.
(j) Emergency plans for rescue medication, medication-related concerns including drug supply and monitoring.

For the domain social circumstances and support the priority statements were as follows.

(a) Access to social support services.
(b) Social isolation.
(c) Care staff shortages and turnover.
(d) Risk of lack of staff training.
(e) Financial hardship.
(f) Loss of respite care.
(g) Risk of abuse/neglect.
(h) Change in accommodation/breakdown of placements.

## Discussion

Priorities identified can help inform allocation of resources for the COVID-19 pandemic, as well as provide relevant information pertinent to potential future pandemics. They do not encompass all potential challenges posed to the IDD community during COVID-19, but rather highlight priority concerns for this vulnerable group.

There is an urgent need to collect high-quality data on the mental health effects of the COVID-19 pandemic, but this should be guided by the priorities of the IDD community. It is essential that the response to COVID-19 is inclusive of people with IDD, to avoid widening pre-existing disparities for this vulnerable group, whose specific needs are frequently overlooked.^3^ Our findings suggest that the key concerns raised by individuals and organisations including service users, family members and charities in the field are also substantially shared by professionals. The 27 priority statements listed may help guide the direction of COVID-19-related research about people with IDD, as they identify what issues appear to be important in this group. The involvement of individuals with IDD and their carers should be embedded in all stages of the research process, as their lived experience is invaluable.^8^

### Mental health and challenging behaviour

#### Implications for clinical practice

Individuals with IDD may be at greater risk of mental health deterioration during COVID-19, for a variety of factors. These include difficulties accessing services, restrictions brought about by lockdown regulations and as a direct effect of fear and anxiety brought about by the pandemic and related media coverage.^8,9^ An increase in the frequency and/or intensity of challenging behaviours may be similarly observed. COVID-19 has also brought about a substantial change in the routines of the general population, but this may be particularly challenging for people with IDD, especially individuals with autism, who may rely on a strict daily routine to maintain a sense of control over their environment, maintain emotional well-being and minimise occurrence of challenging behaviours.^10^

Perhaps understandably considering concerns over deterioration in the mental health and challenging behaviours of people with IDD, carer strain was also highlighted as a priority concern. Related to this, COVID-19 has also had an impact on the level of social support for people with IDD, including day centre care and respite arrangements, which will subsequently further increase carer strain.^11^

Over-prescription of medication is a long-standing concern among the IDD community. Initiatives such as Stopping Over-Medication of People with a Learning Disability (STOMP)^12^ have been implemented to endeavour to reduce medication burden in this group. During COVID-19, some non-medication-based strategies to support people with IDD, such as accessing the community, may no longer be viable. This may potentially lead to the risk of increasing reliance on medication strategies to support mental health and manage challenging behaviour.

The phenomenon whereby symptoms or behaviours in people with IDD are attributed to their underlying developmental condition rather than effectively exploring potential co-occurring physical or psychiatric conditions, has long been thought to be a substantial obstacle to ensuring this group receive optimal care.^13^ There may conceivably be an increased risk of this phenomenon during COVID-19, considering many consultations taking place via telephone or video-conference as opposed to in person.

#### Implications for policy

IDD professionals should always consider the well-being of those whom care for their patients. This is especially true during COVID-19, where timely professional intervention could prevent progression to the detrimental sequelae associated with carer strain, including reduced psychological well-being^14^ and self-esteem,^15^ as well as chronic stress.^16^

It is essential that medication is only prescribed where all other viable non-medication-based strategies have been explored.^17^ If there is no alternative to prescribe medication for challenging behaviour, it is imperative that the rationale is clearly documented. The response to medication should be regularly reviewed, with a view to discontinuing as soon as safe to do so, in keeping with national guidance.^17^

Difficulties in understanding COVID-19 and the related guidance can be addressed through a number of strategies. These include healthcare professionals providing explanations to people with IDD during consultations, tailored to the individual's developmental level, as well as provision of accessible information designed for this group, such as that developed by MENCAP.^18^

People with IDD may find the changes in consultations with healthcare professionals particularly challenging, including remote consultations via telephone or video-link, as well as professionals wearing personal protective equipment. A recommendation has been made to include several strategies to mitigate these challenges, such as slowing down your speech, using a positive tone of voice, affixing a photograph to your clothing and avoiding complex sentences.^19^

The risks of assuming that an individual's difficulties are because of their IDD can be mitigated via thorough enquiry by healthcare professionals. They should also be prepared to undertake face-to-face consultations where diagnostic uncertainty exists, while strictly adhering to relevant infection control rules.

#### Implications for research and service development

Areas for future research should focus on approaches to measure and facilitate access to mental health services. The quality of such care should also form a key focus, including clinicians’ prescribing behaviours, as well as the quality of communication within and outside of consultation settings. Research into carer experiences can also help further delineate how the lived experience of the COVID-19 pandemic can contribute to carer strain, helping develop evidence-based strategies to address this issue.

### Physical health and epilepsy

#### Implications for clinical practice

The consensus statements identify the physical health and epilepsy-related concerns in the IDD community. As understanding of COVID-19 evolves, there is a need to consider how this has an impact on those with IDD. The risks are likely elevated for a section of the IDD population based on the spectrum of comorbid medical conditions, with increased risk of poor health outcomes.^5^

The most frequent concern in the physical health domain, expressed by the stakeholder community, was access to physical healthcare including emergency and routine care.

Emergency care is a source of difficulty for individuals, families and carers of those with IDD needing physical health evaluations, which has long been recognised. Barriers include discrimination by staff and diagnostic over-shadowing.^20^ Numbers attending emergency departments in the UK dramatically reduced during the surge phase of the pandemic.^21,22^ There is a sense in the stakeholder community that fear and a sense of feeling unwelcome could result in delayed presentations of people with IDD.

The physical complications of COVID-19 including risk of atypical presentations of physical illness that may go initially unrecognised are of concern. The impact of COVID-19 in IDD, which could result in worsening seizure control in individuals with poorly controlled epilepsy, is highlighted by the importance of controlling fever in Dravet syndrome.

There are concerns about the medication care plans, as well as drug supply. Given that people with IDD and epilepsy may have an increased risk of status epilepticus, the importance of rescue medication plans cannot be understated.^23^ This may terminate clusters and prolonged life-threatening seizures and potentially reduce risk of hospital admission and the possibly intensive care admission. Drug supply was raised as a concern related to COVID-19, and this did not seem to present itself as a major problem in terms of overall drug supply lines.^24^

There is particular concern that people with IDD would face discrimination in end-of-life care, with ventilator or intensive care unit bed resource allocation decisions made on the basis of IDD, which is considered a morally irrelevant factor by some.^25^

#### Implications for policy

Sufficient community resources, including specialist nursing teams, need to be sustained to prevent spikes in avoidable admissions. This should be factored into any decisions for staff redeployment.

The pandemic has meant routine healthcare is now largely delivered by telephone or video consultations.^26^ Lack of physical health reviews on a routine basis was a concern as they identify unmet needs and implement interventions to improve health outcomes. Epilepsy care has been conceivably adversely affected by the lack of face-to-face consultations in certain situations. Vagus nerve stimulation assessments, physical exams, assessing weight, dental checks,^27^ as well as assessing biochemical and nutritional variables (for ketogenic diet) have all been significantly more difficult to coordinate.^28,29^ Monitoring risk in IDD populations from seizure-related harms and sudden unexpected death in epilepsy is possible by telemedicine methods using validated checklists.^30^ There has been a lack of access to electroencephalogram diagnostic testing in many regions, as well as epilepsy surgical programmes having to temporarily suspend activity.

Telemedicine has advantages in that people who cannot get to clinic or do not like travelling can be reviewed, as can various carers or family members all simultaneously.^29^ The disadvantage, however, lies in the lack of preparation and training in video assessments, which may result in poor engagement.

Identifying those at highest risk from COVID is challenging as IDD is not homogeneous and this was acknowledged by the stakeholders. Making recommendations about shielding is challenging; adoption of governmental guidelines is an adequate approach in the absence of more specific evidence.^31^ Most expert groups have suggested people with epilepsy for the most part will not have elevated risk, and few reports have been published suggesting acute symptomatic seizures in the context of COVID-19.^32^ Some provisional and methodologically limited Spanish data has, however, suggested otherwise.^33^

This survey has highlighted concerns over a lack of opportunity and appropriate supervision and assistance for those with IDD to participate in exercise. The lack of physiotherapy support was mentioned. The sedentary behaviour and risk of weight gain could be particularly adverse for individuals if they develop COVID-19 and attempts to maintain physical fitness should be endorsed. Group online solutions should be considered for feasibility. This also applies to people with epilepsy, who have been reported to have higher rates of sedentary behaviours.^34^

Healthcare settings across the UK reported shortages of personal protective equipment, and this was a concern of our stakeholder group. This is important in private care facilities and National Health Service hospital settings. The ability of people with IDD to comply with use of masks for personal safety may be problematic, which may limit socialising opportunities in the time of emergence from the pandemic surge.

Discrimination pertaining to end-of-life care decisions for people with IDD is likely to be subtle and covert.^35^ It is to be encouraged that such decisions should be made by a multidisciplinary team, including individuals with expertise in supporting and advocating for the needs of individuals with IDD. Practitioners should strongly consider advanced care planning where appropriate.

#### Implications for research and service development

Research and service development work should focus on the experiences and outcomes of individuals with IDD when requiring emergency care during the pandemic, for complications of COVID-19 infection, as well as unrelated physical health complaints. Healthcare professionals need to measure physical-health-related outcomes during the COVID-19 pandemic. These include seizure frequency, admissions and mortality rates, to understand the level of difference in such outcomes compared with pre-COVID-19. Informed recommendations on how to address any identified increased risks need to be developed. Pandemic-friendly exercise interventions can be trialled, to establish how they compare with alternative approaches, or indeed, a lack of exercise.

For individuals with IDD who become severely unwell requiring intensive care unit admission and/or a ventilator, their experiences and outcomes can be compared with their non-IDD peers who become similarly unwell. This will ascertain if there is a difference regarding end-of-life care quality, and if so, develop strategies to ensure effective advocacy for people with IDD.

### Social circumstances and support

#### Implications for clinical practice

As for mental and physical health services, concerns are also reported for social support services. The impact of reduced day centre care and professional carer support during the COVID-19 pandemic also presents a substantial challenge to the IDD community.

Members of the IDD community may feel increasingly socially isolated during the pandemic. They may no longer be able to access their regular day centre care, job or social circle, at least not in person, with a potentially detrimental impact on their well-being.

Concern about care staff shortages and turnover during COVID-19 has previously been reported.^36^ It is essential that any substitute caregiver is suitably educated about the individual needs of the person with IDD whom they are caring for, to minimise the impact of such a change. Even with optimal transitions of care, however, there may be a detrimental impact on the individual with IDD, as previous findings have shown continuity of care to be significantly associated with their quality of life.^37^ This is linked to concerns that staff may be inadequately trained, as well as concerns of a lack of COVID-19-specific training for care staff. COVID-19-specific training can be provided locally, as well as utilising freely available online training, such as that provided by the World Health Organization.^38^

Loss of respite care during COVID-19 has a detrimental effect on carers of people with IDD, who may have benefited substantially from such breaks in care with regard to their mental health and overall well-being.^39^ Individuals with IDD may also suffer from the consequences of a loss of respite care, such as from the disruption to their routine from not attending or indirectly from their regular carer's impaired ability to care for them as a result of increased carer strain.

People with IDD may be especially vulnerable to abuse during the COVID-19 pandemic as usual community-based support sources (such as family members and mental health professionals) may not be available because of social distancing.^39^

Some individuals with IDD may experience a change in their accommodation during COVID-19. This may be moving from a more crowded residential care setting back into the family home, with a view to reducing likelihood of infection. This may, however, have unintended detrimental consequences resulting from the departure from familiar routine for the individual with IDD, as well as the family having a lack of experience in caring for them for prolonged periods of time. Equally, care home placements may breakdown during the COVID-19 pandemic, likely as a consequence of a multitude of factors, including deterioration in mental health and challenging behaviours, disruption of routine, high staff turnover and social isolation, among others. This concern has been previously raised.^39,40^ IDD professionals have a collective responsibility to address proactively such situations and prevent placements where people under their care had been previously functioning well from collapsing.

#### Implications for policy

Concerns have previously been reported about professionals working in the IDD sector potentially being redeployed to other services during the pandemic.^41^ We share these concerns, as even prior to COVID-19, people with IDD were a substantially marginalised group who experienced significant barriers to mental, physical and social support. It is imperative that they do not experience further such discrimination at a time when their support needs are likely to be substantially increased.^41^

Digital communication presents a crucial tool in maintaining contact with loved ones, and people with IDD need to be supported to ensure that they can use such technology in order to maintain crucial relationships during the pandemic.^42^

There are understandable concerns about financial hardship for people with IDD during COVID-19, as well as for their care providers. For patient-facing third-sector organizations such as charities, particularly those that represent vulnerable and marginalised groups like those with intellectual disability, the World Health Organization recommend that the government provide sustainability grants to help continue their core work of representation of the groups they support.^38^

#### Implications for research and service development

Areas for further study in this aspect of care should focus on the experiences of people with IDD during the pandemic, particularly related to social isolation, with a view to developing strategies to maintain social inclusion during such challenging circumstances. One option may be to explore virtual technologies as a means of enabling groups of people with IDD to communicate with one another and/or with carers remotely. This could include videotelephony; as such services have received positive feedback in focus groups for people with IDD, who report an increased sense of security and safety.^43^ Handover processes for carers should also be reviewed, to ensure that people with IDD are being cared for by individuals with an in-depth knowledge of their specific care needs.

Pandemic-related factors that likely contribute to placement breakdown, such as loss of respite care and high staff turnover should be researched thoroughly. This is required to establish what strategies can be employed in a pandemic situation to rescue placements where persons with IDD were previously functioning well. Additionally, where different forms of financial support for carers and persons with IDD are given, these can be analysed and compared to establish their efficacy in improving/maintaining the quality of lives of the recipients. This should also be used to establish what approach brings about the greatest reward per unit of investment.

### Limitations

Members of the IDD community as well as their corresponding organisations were almost all based in England (29 of the 32 organisations invited to take part). As a result, one cannot be certain as to whether the priority concerns identified here are generalisable to the UK, or indeed from an international perspective.

A further limitation is that the expert panel solely comprised healthcare professionals. Ideally, individuals with IDD and carers should have also been part of this panel, to ensure representation in all stages process, and avoid the potential for healthcare professionals to have too great an influence over the development of the resulting consensus statements.

## Data Availability

The data that support the findings of this study are available from the corresponding author upon reasonable request.

## Supplementary material

For supplementary material accompanying this paper visit https://doi.org/10.1192/bjo.2020.122. click here to view supplementary material

## Appendix

### List of responding organisations

**Table:**

Organisation
Alder Hey Children's Hospital Ancora Medical Practice Association of Chartered Physiotherapists for People with Learning Disabilities British Dietetic Association Child and Young Person's Clinical Psychology Network Cork University Hospital Epilepsy Action Epilepsy Ireland Epilepsy Nurses Association Epilepsy Society Epilepsy Sparks International League Against Epilepsy Learning Disability Professional Senate Medicines Safety in Neurodiversity Pennine Care NHS Foundation Trust Radiant Research Consortium Royal College of General Practitioners Royal College of Occupational Therapists, Specialist Section, People with Learning Disabilities Royal College of Psychiatrists Royal College of Speech and Language Therapists Royal Mencap Society Royal Society of Medicine SUDEP Action Swansea Bay UHB UCL Queen Square Institute of Neurology, Department of Experimental & Clinical Epilepsy Young Epilepsy
